# Clinicopathological and molecular genomic features of monomorphic epitheliotropic intestinal T-cell lymphoma in the Chinese population: a study of 20 cases

**DOI:** 10.1186/s13000-021-01173-5

**Published:** 2021-12-12

**Authors:** Chunni Chen, Yuxi Gong, Yefan Yang, Qiuyuan Xia, Qiu Rao, Yang Shao, Liuqing Zhu, Junli Zhang, Xiao Li, Pan Ji, Boya Zhai, Xiang Zhang, Zhihong Zhang

**Affiliations:** 1 Department of Pathology, the First Affiliated Hospital of Nanjing Medical University, 300 Guangzhou Road, 210029 Nanjing, Jiangsu Province China; 2 Department of Pathology, Nanjing Jinling Hospital, Nanjing University School of Medicine, 210002 Nanjing, Jiangsu Province China; 3 Nanjing Geneseeq Technology Inc, 210032 Nanjing, Jiangsu Province China; 4 School of Public Health, Nanjing Medical University, 211166 Nanjing, Jiangsu Province China

**Keywords:** Monomorphic epitheliotropic intestinal T-cell lymphoma, Whole-exome sequencing, JAK-STAT pathway, Amplification of chromosome 9q

## Abstract

**Background:**

Monomorphic epitheliotropic T-cell lymphoma (MEITL) is an aggressive non-Hodgkin lymphoma with a high fatality rate. This study was aimed to explore the clinicopathological and molecular genetic features of MEITL in the Chinese population.

**Methods:**

A retrospective analysis was performed based on the clinical manifestations and pathological features of 20 Chinese MEITL. 9 cases with paired diseased-normal tissues were also analyzed for molecular information by whole-exome sequencing.

**Results:**

There were 14 men and 6 women with a median age of 58.5 (28-81) years. 17(17/20) lesions were located in the jejunum or ileum; 13(13/20) cases had ulcers or perforations. Microscopically, except for 1(1/20) case of pleomorphic cells, the monomorphic, middle-sized tumor cells infiltrating into the intestinal epithelial and peripheral intestinal mucosa recess could be seen in the other 19 cases. Immunohistochemistry showed that most of the tumor cells in MEITL were positive for CD3(20/20), CD8(17/20), CD43(19/20), and CD56(15/20), but negative for CD5(20/20). The most frequently mutated genes of these Chinese cases were *STAT5B* (4/9) and *TP53* (4/9), not *SETD2*(2/9). *JAK3* mutations (3/9) were also detected with a high mutated frequency. We demonstrated that mutations of JAK-STAT pathway-related genes and the amplification of Chromosome 9q appeared at the same time in most cases(5/9).

**Conclusions:**

The clinicopathological features were consistent with that in previous western studies, but a special case with pleomorphic cells was found in this study. The co-occurrence of JAK-STAT pathway-related gene mutations and the amplification of Chr9q is a molecular feature of MEITL.

**Supplementary Information:**

The online version contains supplementary material available at 10.1186/s13000-021-01173-5.

## Introduction

Monomorphic epitheliotropic intestinal T-cell lymphoma (MEITL) is an aggressive intestinal non-Hodgkin lymphoma originating from T cells, which has been classified as a separate category from enteropathy-associated T-cell lymphoma (EATL) in the 2016 WHO classification of hematopoietic and lymphoid tissues [1]. MEITL is more common in Asia with monomorphic CD3+ CD8+ CD56+ tumor cells and lacks clinical evidence of celiac sprue [2]. Frequent mutations in *SETD2*, which encodes a non-redundant H3K36-specific trimethyltransferase, have been found in most MEITL cases in several studies, and the JAK/STAT pathway is also reported to be the most commonly mutated signaling pathway [3–5].

Due to its low incidence, and a lack of researches on large-scale cases, the clinicopathological and molecular characteristics of MEITL in the Chinese population have not been fully clarified. The present study performed a retrospective analysis based on clinicopathological features of 20 MEITL cases, nine of which also underwent whole-exome sequencing for molecular genetic information, to provide a basis for clinical diagnosis and treatment and supplement the genetic landscape of MEITL in the Chinese population.

## Materials and methods

### Sample selection

The use of samples in this study was in accordance with the Declaration of Helsinki and approved by the Ethics Committee of The First Affiliated Hospital of Nanjing Medical University (2020-SR-169).

Patients were eligible for study inclusion if they: (1) were comprehensively diagnosed with MEITL. (2) had complete clinical data. (3) had undergone surgical resection between 2013 and 2019. According to the WHO classification of Tumors of Hematopoietic and Lymphoid Tissue (2016), the diagnostic criteria of MEITL were as follows: (1) Patients had no association with coeliac disease. (2) The monomorphic medium-sized neoplastic lymphocytes showed prominent epitheliotropism. (3) The tumor was aggressive in behavior. (4) The tumor expressed CD3, CD8, CD56, but not CD5.

Fifteen MEITL cases from The First Affiliated Hospital of Nanjing Medical University and 5 MEITL cases from Nanjing Jinling Hospital met the inclusion criteria. Hematoxylin-eosin staining and immunohistochemical slides were retrieved and re-evaluated by two pathologists (Z. Zhang and C. Chen) who had specific hematopathology training. Nine cases with enough paired diseased-normal tissues were selected for whole-exome sequencing.

### Clinical and pathological indicators

Patients’ general information, clinical manifestations, pathological morphology, were collected for subsequent analysis. The general information and clinical manifestations include age, gender, locations, symptoms (bloating, abdominal pain, fever, anemia, diarrhea, abdominal mass), complications (intestinal obstruction, perforation). The laboratory data covered C-reaction protein (CRP), serum albumin, high-density lipoprotein, low-density lipoprotein and hemoglobin. Since all 20 patients had no typical symptoms of celiac disease such as steatorrhea or gluten intolerance before diagnosis, and most of them were suspected of inflammatory bowel disease or acute appendicitis, specific serologic testing for celiac disease was not performed. The pathological morphology focused on the following aspects: gross morphology, cell morphology, growth pattern, infiltration depth, epitheliotropic pattern, neoplastic necrosis, mitosis, inflammation, crypt changes in adjacent mucosae and lymph node involvements.

### Immunohistochemistry

Immunohistochemical staining was performed on 3 μm thick formalin-fixed paraffin-embedded tissue sections using Ventana automated (BenchMarker) stainer (Tucson, Roche, USA) according to the manufacturer’s instructions. Standard immunohistochemical studies were performed using the following antibodies: CD2, CD3, CD4, CD5, CD7, CD8, CD15, CD20, CD30, CD43, CD56, TIA-1, Granzyme B, perforin, S-100, EMA, Syn, Bcl-2, Bcl-2, ALK, Ki-67. All antibodies and EBER probes were ready to use and bought from Maxim biotechnology development co. LTD (China).

Immunohistochemistry results were scored according to the intensity of staining and the percentage of positive cells. The IHC results of all cases were interpreted by two pathologists using a double-blinded method, and the Ki-67 index only evaluated the percentage of positive cells.

### Whole-exome sequence

Genomic DNA was firstly extracted from selected FFPE surgical samples of 9 MEITL cases using QIAamp DNA FFPE tissue kit (Qiagen). To prepare materials for constructing the DNA library, we fragmented 2 µg DNA using Covaris M220 sonication system (Covaris), followed by end-repairing, A-tailing, and adaptor ligation and purification by KAPA Hyper Prep Kit (KAPA Biosystems). What’s more, the selected DNA fragments needed to be further amplified and purified before the Illumina Rapid Capture Extended Exome Kit (Illumina Inc.) could be used for exome enrichment. Finally, captured libraries were submitted to the Illumina Hiseq 2500 platform (Illumina Inc.) for further sequencing. The sequenced samples passing the following quality control criteria were subject to later sequence analysis: (1) DNA extracted from each sample ≥50ng with A260/280 ranged from 1.8 to 2.0; (2) DNA in the pre-library >100ng; (3) Q30 (the percentage of bases with mass value ≥30) >75%; (4) mean sequencing depth of 150× for tissues and 60× for negative control tissues.

### Sequence analysis and pathway enrichment analysis

Trimmomatic was performed on FASTQ file to remove leading/training low quality(quality reading below 20) or N bases for quality control. Paired-end sequencing data after quality control was aligned to the human reference genome hg19 using Burrows-Wheeler Aligner (BWA-MEM)[6], followed by sorting, PCR-duplicate removal, base quality score recalibration(BQSR) using Samtool v1.6, Picard v1.119 (http://picard.sourceforge.net/) and Genome Analysis Toolkit (GAKT)[7], respectively. VCF2LR (GeneTalk) was used to check the same single nucleotide polymorphism (SNP) fingerprint in paired tumor and normal sample, and then, nonmatching samples or samples with a mean depth <30X were removed from the analysis. Cross-sample contamination was estimated using ContEst (Broad Institute). Single nucleotide variants (SNV) and germline mutations were identified by a useful variant caller — VarDict-1.5.4 [8], and Scalpel (http://scalpel.sourceforge.net) was an effective algorithm for mining insertion and deletion mutations [9]. SNVs and indels identified were filtered if: 1) presented >1% frequency in the 1,000 Genomic project; 2) variant-supporting reads < 4 or variant allele frequency (VAF) < 2%; 3) caused by sequencing or genome bias, and presented in multiple or all samples in the same batch at the same time. The final annotations of all SNVs and indels were acquired using vcf2maf-1.6.16.

Copy number variants (CNVs) were calculated using FACETS [10] after data purification. According to the total copy number (tcn) of each genomic segment and sample ploidy, CNV events could be divided into three types: amplification ((tcn-ploidy) ≥1); neutral (-1< (tcn-ploidy) <1); deletion ((tcn-ploidy) ≤-1). If at least 60% of the segments on a chromosome had a consistent CNV level, this event was assessed at the chromosome level. For focal CNV events, deep amplifications and deep deletions were counted for further analyses. Gene-level CNVs were identified with the log2 depth ratio of >0.6 for copy number gain and <-0.6 for copy number loss.

We performed Gene Ontology (GO, http://www.geneontology.org/) and Kyoto Encyclopedia of Genes and Genomes (KEGG, https://www.kegg.jp/) analysis to explore the biological processes and pathways associated with MEITL.

### Prediction of mutations on protein stability

Polyphen2 (Polymorphism Phenotyping v2), SIFT (Sorting intolerant of tolerant substitution) are used to predict the possible impact of an amino acid substitution on protein structure by generating a score based on each sequence. The default score threshold of SIFT is currently set at 0.05 for binary classification (deleterious: <0.05; tolerated: ≥0.05) with a balanced accuracy of 76.99% (http://sift.jcvi.org/) [11]. The score in Polyphen2 ranges from 0 to 1, and the smaller the score, the less likely it is to damage protein stability. According to the score, mutations are divided into benign (0-0.452), possibly damaging (0.447-0.909) and probably damaging (0.909-1) (http://genetics.bwh.harvard.edu/pph2/)[12].

### Therapy and follow-up

Therapies and outcomes were obtained from electronic medical record system or telephone interviews, and the deadline for follow-up was October 2021. The overall survival (OS) was defined as the time interval from the date of diagnosis to the end of follow-up or death.

## Results

### Clinical findings

There were 14 men and 6 women with a median age of 58.5(range, 28-81 years). Among all cases, 17(17/20) lesions were located in the jejunum or ileum, 2(2/20) in the colon, and 1(1/20) in both ileum and colon. Patients had common gastrointestinal symptoms, including anemia (19/20), abdominal pain (17/20), and intestinal perforation (10/20). Weight loss (8/20), abdominal mass (7/20), fever (6/20), bloating (6/20), diarrhea (3/20), and intestinal obstruction (3/20) were observed in less than half of the cases. All patients had no evidence of celiac disease or inflammatory bowel disease. Hypoalbuminemia, low high-density lipoprotein level, and elevated CRP level were detected in 14 (14/20), 18(18/20), and 16(16/20) cases, respectively. While no cases had elevated low-density lipoprotein level.

### Pathological features in MEITL

The main morphologic and immunophenotypic features of 20 MEITL cases were shown in Table 1.

**Table 1:** The main clinicopathological features and outcomes of 20 MEITL cases.

case	Sex	Age	Location	Grossly	Cell morphology	Infiltration depth	lymph node involvements	CD3	CD5	CD4	CD8	CD56	Postoperative treatment	Outcomes(months)
1	M	51	small intestine	perforation	M	Full-thickness	N	+	-	-	+	+	NA	D(6.5)
2	M	56	small intestine	mass	M+P	Full-thickness	Y	+	-	-	+	+	CHOP	D(24)
3	M	69	small intestine	perforation	M	Full-thickness	N	+	-	-	+	+	CHOPE	D(9)
4	F	59	small intestine	mass	M	Full-thickness	N	+	-	-	+	+	CHOP+ASCT	D(9)
5	F	81	small intestine	perforation	M	Full-thickness	N	+	-	-	+	+	NA	D(0.5)
6	M	58	small intestine	ulcer	M	Mucosa and submucosa	N	+	-	-	-	+	CHOP	D(7.5)
7	M	28	small intestine	mass	M	Full-thickness	N	+	-	-	+	-	CHOPE	D(7)
8	M	35	small intestine	perforation	M	Full-thickness	N	+	-	-	+	+	NA	D(0.5)
9	M	63	small intestine	mass	M	Full-thickness	N	+	-	-	+	+	DA-CHOPE	D(9)
10	M	66	small intestine	perforation	M	Full-thickness	Y	+	-	+	+	-	NA	D(0.5)
11	M	52	small intestine	perforation	M	Full-thickness	N	+	-	-	+	+	CHOPE	D(56)
12	F	59	small and large intestine	mass	M	Full-thickness	N	+	-	-	-	-	DA-CHOPE	D(58)
13	M	54	small intestine	perforation	M	Full-thickness	Y	+	-	-	+	+	CHOP	D(6)
14	M	70	small intestine	perforation	M	Full-thickness	N	+	-	-	+	-	DA-CHOPE	A(101)
15	F	46	large intestine	mass	M	Mucosa and submucosa	N	+	-	+	+	+	CHOPE+ASCT	D(10)
16	M	60	small intestine	perforation	M	Full-thickness	N	+	-	-	+	+	DA-CHOPE+ASCT	D(6)
17	M	81	large intestine	mass	M	Full-thickness	N	+	-	-	+	-	Mini-CHOP2	D(26)
18	M	71	small intestine	perforation	M	Full-thickness	N	+	-	-	+	+	CHOPE	NA
19	F	51	small intestine	ulcer	M	Full-thickness	N	+	-	-	+	+	CHOPE	D(12.5)
20	F	39	small intestine	perforation	M	Full-thickness	N	+	-	-	-	+	NA	D(1.5)
No (%)								20 (100)	0 (100)	2 (10)	17 (85)	15 (75)		

Sex: M-male, F-female; Cell morphology: M-monomorphic, P-pleomorphic; lymph node involvements: Y- with lymph node involvements, N-without lymph node involvements; Postoperative treatment: NA- with no treatment, C-cyclophosphamide, H- adriamycin, O- vincristine, P-prednisone, E-etoposide, ASCT- autologous hematopoietic stem cell transplantation; Outcomes(months): D- dead, A- alive, NA- lost to follow-up

Seventeen jejunoileal specimens, 1 ileocolic specimen, and 2 colorectal specimens were selected for examination. Grossly, thirteen (13/20) cases showed single/multiple ulcers and perforations, and the mucosal folds around the rupture disappeared, part of the serous membrane had fibrinopurulent exudates. Seven (7/20) cases developed solid masses of size ranging from 3.2 cm*2 cm*1.3 to 10 cm*4 cm*1 cm with grayish-white and reddish cut surface, infiltrating the full- thickness of the intestinal wall.

**Fig. 1:**
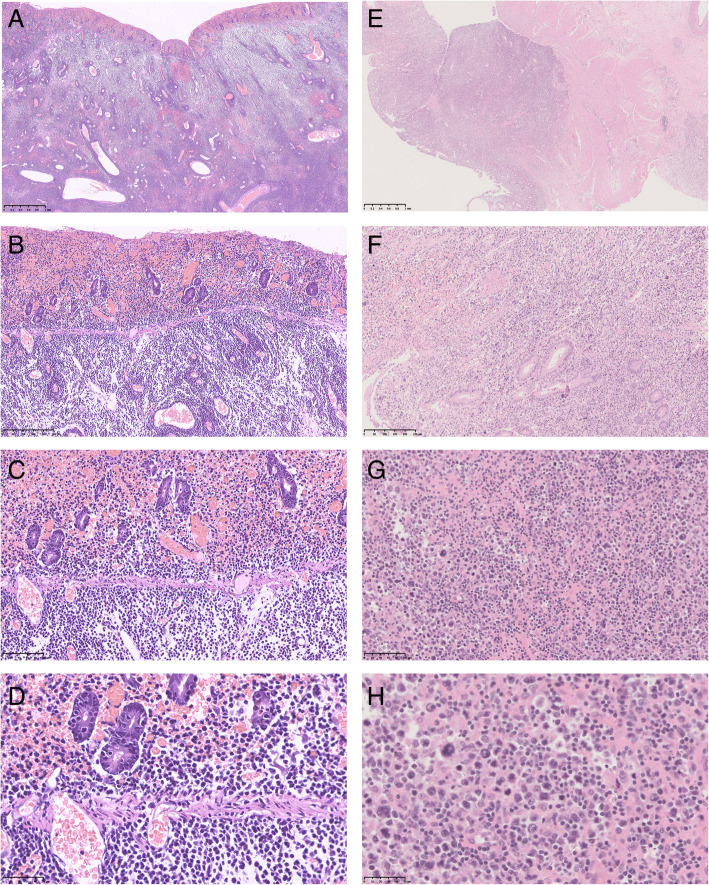
The morpholgic features of monomorphic epitheliotropic intestinal T⁃cell lymphoma. A, E: ×2; B, F: ×10; C, G: ×20; D, H: ×40. **A-D** epitheliotropic, monomorphic medium-sized tumor cells with round dark nuclei and pale cytoplasm; (E-H) epitheliotropic, polymorphic cells with anaplastic characteristics (large nuclei, obvious nucleoli, increased mitotic activity and nuclear fragmentation) scattered among monomorphic medium-sized cells

Microscopically, most tumor cells were monomorphic medium-sized cells with round dark nuclei and pale cytoplasm (Fig. 1 A-D), spreading to adjacent areas and infiltrating the full-thickness of the intestinal wall. Epitheliotropic pattern (Fig. 1 A, E) was demonstrated in all 20 cases, with obvious neoplastic necrosis at the perforation and scattered acute and chronic inflammatory cells in the interstitium. Villus atrophy, crypt hyperplasia, and increased lymphocytes in the crypt and surface epithelium were not found in adjacent mucosae. Lymph node involvements were histologically confirmed in 3 (3/20) cases.

**Fig. 2:**
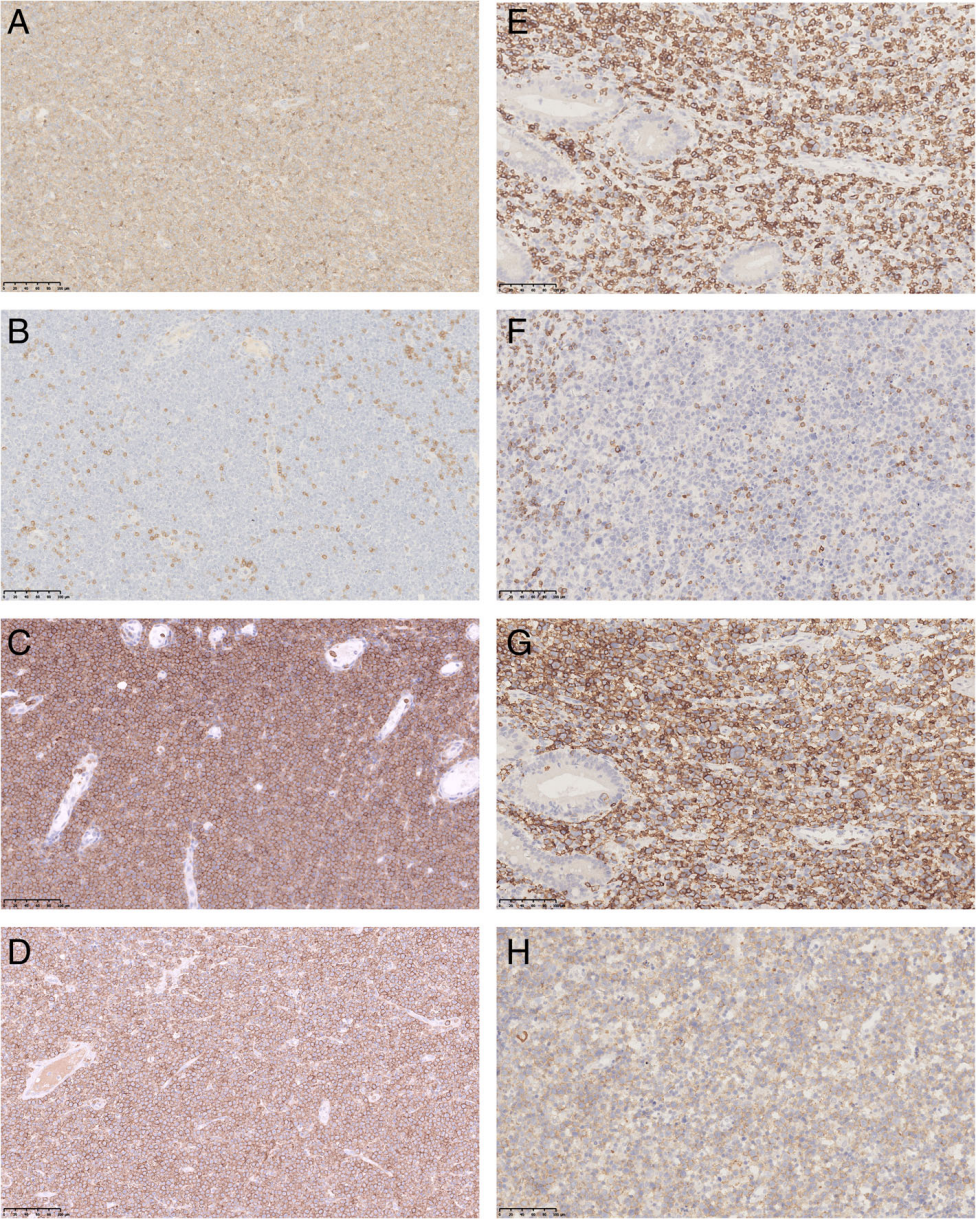
The immunophenotypes of monomorphic epitheliotropic intestinal T⁃cell lymphoma. A-H: ×20. A-D: MEITL with diffuse monomorphic cells. E-H: MEITL with scattered pleomorphic cells. **A, E** CD3+; (**B, F**) CD5-; (**C, G**) CD8+; (**D, H**) CD56+

Tumor cells in all 20 cases were strongly positive for CD3 (Fig. 2 A, E) and CD7, while CD2(19/20), CD43(19/20), CD8(17/20) (Fig. 2 C, G), and CD56(15/20) (Fig. 2D, H) were positive for partial tumor cells. CD4(2/20) and B cell markers, like CD20(1/20) and CD79α(1/20) were positive in scattered tumor cells; whereas CD5(Fig. 2B, F) was negative in all cases. One case was double negative for CD8 and CD56. The Ki-67 index was generally high ranging from 60 to 90%. Interestingly, more than half of these cases expressed cytotoxic markers including TIA-1(19/20), Granzyme B(12/20), and perforin(11/20). Moreover, all cases were EBER-ISH negative.

It was worth noting that there was an interesting case that had co-occurrence of both monomorphic and pleomorphic regions. The pleomorphic region showed large pleomorphic tumor cells with anaplastic characteristics (large irregular nuclei, obvious nucleoli, increased mitotic activity and nuclear fragmentation) scattered among monomorphic medium-sized cells (Fig. 1E-H). Multinuclear cells and giant cells were also seen. Although most areas of the epithelium were lost due to the ulcer, prominent epitheliotropism could also be seen. Both monomorphic and pleomorphic neoplastic lymphocytes were positive for CD3 (Fig. 2E), CD8 (Fig. 2G), CD56 (Fig. 2 H), TIA-1, Bcl-2, and negative for CD4, CD5 (Fig. 2 F), ALK, CD20, CD15, CD30, S-100, EMA, Syn, Bcl-6. The Ki-67 index was about 70%.

### Molecular genetic findings

The main Molecular genetic findings of 9 MEITL cases were shown in Table 2 and supplementary tables.

**Table 2:** The main genetic findings of 9 MEITL cases.

case	Somatic mutations					Copy number variations	
	STAT5B	TP53	JAK3	SETD2	STAT5A	chromosomal arms	CNV type
1		p.R248Q	p.M511I		p.N642H p.G472S	8p,8q,9p,12p,20p	deletion
						4q,7q,9q	amplification
2	p.N642H	p.I232Sfs*15				4p,4q,7p,10p,10q,15q,18p	deletion
						1q,9q,19q	amplification
3	p.Y665F	p.V147Afs*19	p.A573V	p.E1772_I1781delinsV p.D2004Ifs*13		4p,7p,8p,18p,21q	deletion
						7q,8q,9q,12p,12q,18q,22q	amplification
4					p.Q701L	6p,7p,18p	deletion
						9q,21q	amplification
5						7p,8p	deletion
						7q,9q,19q,19p	amplification
6	p.A766V	p.G105C	p.R657Q			1q,7q,9q,19q,19p	amplification
7	p.N642H			p.S1572*			
8							
9						21q	deletion
						1q,6p,8q,9q,20q,22q	amplification

**Fig. 3:**
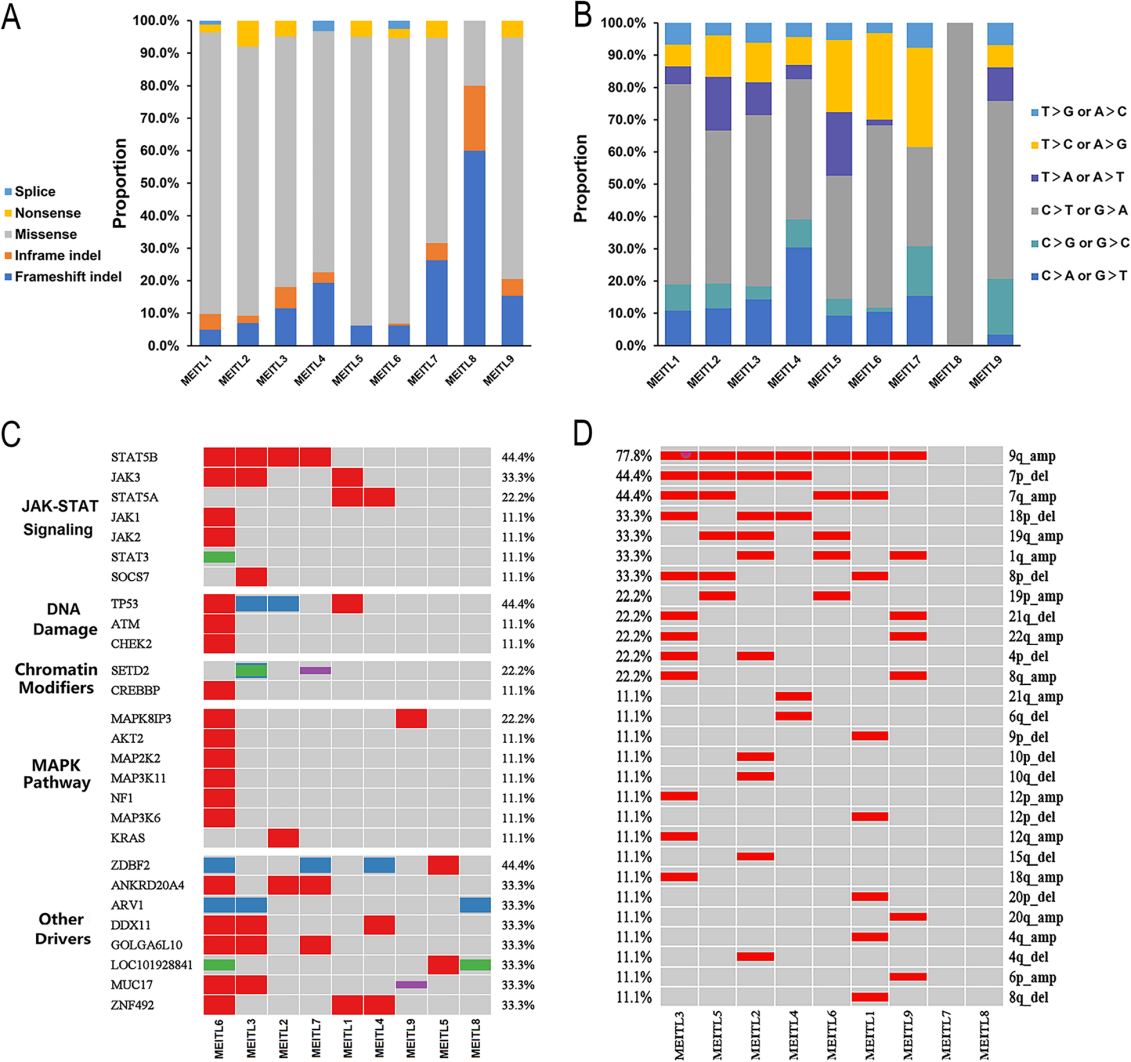
MEITL mainly consisted of missense mutations (**A**) and had a predominance of C>T and G>A transitions (**B**); (**C**) depicted the heat map and associated signal pathways of MEITL, and 66.7% of cases had mutations in genes of JAK-STAT signaling pathway, including *STAT5B*, *JAK3* and *STAT5A*; (**D**) showed the major copy number variants in MEITL, and the most significant chromosome copy number variation was the amplification of 9q, followed by gains of 7q and losses of 7p

**Fig. 4:**
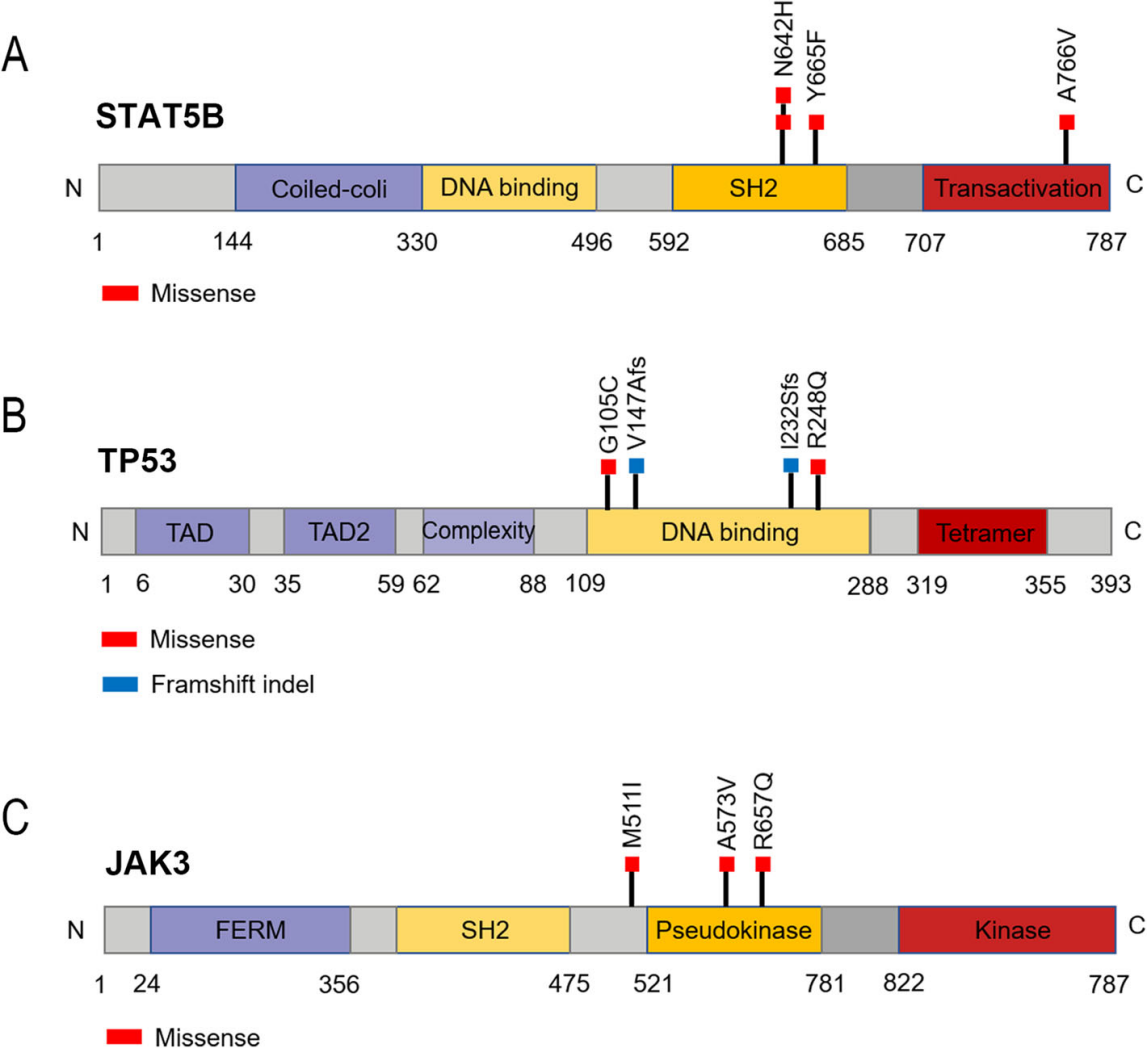
Somatic alterations in *STAT5B*, *TP53* and *JAK3* identified in this study. **A** Four *STAT5B*-mututed cases harbored three distinctive missense variants, including the common N642H mutation(rs938448224) and two mutations—Y665F(COSM1716592) and A766V(COSM8916674); (**B**) Four *TP53*-mututed cases harbored two frameshift variants - V147Afs*19 and I232Sfs, and two missense variants - R248Q and G105C. **C** Three *JAK3*-mututed cases harbored three missense variants - M511I(rs752661478), A573V(COSM34215) and R657Q(rs758959409)

There were 1987 somatic mutations with a median of 220 (range 19-1577) mutations per patient (average of 1.82 per Mb), typically consisting of missense mutations(Fig. 3 A), and the mutated spectrum showed a predominance of C>T and G>A transitions (Fig. 3B). 66.7% of cases had mutations in genes of the JAK-STAT signaling pathway which was known as a major oncogenic mechanism in T cell lymphomas, including *STAT5B* mutations (4/9), *JAK3* mutations (3/9), and *STAT5A* mutations (2/9) (Fig. 3 C). Interestingly, the *STAT5A* mutation and *STAT5B* mutation were found to be mutually excluded, although either could active the JAK-STAT pathway. The four *STAT5B*-mutated cases harbored three distinctive missense variants, including the common N642H mutation(rs938448224) and two mutations—Y665F(COSM1716592) and A766V(COSM8916674) which were only reported in COSMIC (Fig. 4 A). *TP53* variants were discovered in 4 of 9 cases, and all were located in the DNA-binding domain (Fig. 4B). Two of them were frameshift mutations, V147Afs*19 and I232Sfs*15, which were expected to confer critical changes in protein structure, and the other two missense mutations (R248Q and G105C) were predicted to be deleterious with a damaging effect on the protein by SIFT and Polyphen2 algorithms. *JAK3* mutated in 3 (3/9) cases, harboring activating mutations — M511I(rs752661478), A573V(COSM34215) and R657Q(rs758959409) which had been reported frequently in MEITL [3, 13] (Fig. 4 C). Only 2 (2/9) cases had *SETD2* mutations, including two novel frameshift mutations- E1772_I1781delinsV and D2004Ifs*13 in one case and a novel nonsense mutation-S1572* in another case. *JAK1* mutation, *STAT3* mutation, *KRAS* mutation and *GNAI2* mutation were observed in one case (1/9) respectively. And no cases had *BRAF *mutations or *NRAS* mutations.

KEGG and GO enrichment analysis revealed that mutated genes expressed higher in MEITL were enriched for the JAK-STAT signaling pathway and DNA damage system (Fig. 3 C).

Copy number analysis derived from WES data identified multiple regions of frequent gains and losses (Fig. 3D). The most significant chromosomal arm copy number variation of MEITL was the amplification of 9q, accounting for 77.8% of all cases. Gains of 7q(4/9), 19q(3/9), 1q(3/9) and losses of 7p(4/9), 18p(3/9) and 8p(3/9) were also frequent in MEITL (Fig. 3D). Correspondingly, the genomic profile showed recurrent copy number gains at 9q34(7/9), 19q13(7/9), 7q34(6/9), 7q21(5/9), 5q31(5/9), 8q24(5/9), 1q21(5/9), 2q31(5/9), and losses at13q12(4/9), 18p11(4/9). The changes of gene copy number were consistent with that of chromosome arms. Tumor-associated genes on Chr9q such as *FANCC*, *NTRK2*, *SYK*, and *NOTCH1* were amplified; amplification of *BRAF*, *EZH2*, *MET*, *CDK6* and *HGF* located on Chr7 were also detected. Five(5/9) cases including the one with pleomorphic features had both the amplification of Chr9q and mutations of genes of the JAK-STAT pathway.

Frequent germline mutations were also detected. There were 68,419 germline mutations with a median of 7602 (range 6414-8182) mutations per patient, typically consisting of intragenic mutations. Many kinds of variants were documented in all 9 cases, such as *ACPP* c.*428A>G, *CFAP74* c.2176+745 A>G, *EFCAB4B* c.*90A>G, *LIMD1* c.1409-5_1409-3inv.

### Therapy and follow-up

Five of 20 cases did not receive chemotherapy after surgery. The other 15 patients received cyclophosphamide-, adriamycin-, vincristine-, and prednisone- (CHOP) or CHOP-like chemotherapy, 3 of which were followed by autologous hematopoietic stem cell transplantation(ASCT). During the follow-up, 18 (18/20) patients died, 1 (1/20) was alive, and 1 was lost to follow-up. The median OS was 9(0.5-101) months, with a 3-year OS of 15.8%. The outcome data were shown in Table 1.

## Discussion

The present study analyzed the clinicopathological and molecular genetic characteristics of 20 Chinese patients with MEITL. In line with the previous studies [14, 15], men were more susceptible than women with a ratio of 2:1. MEITL patients commonly displayed primary symptoms of abdominal pain, accompanied by systemic symptoms such as fever, anemia, and weight loss. Complications such as intestinal perforation are also common. MEITL mostly occurred in the small intestine, but could also inflect the whole GI tract[16]. Previous studies reported colon and stomach as primary sites that also involved mesenteric nodules or other distant organs including liver, spleen, lung, bone, or skin [17, 18]. Skin involvement was rare as it only occurred in 5% of all patients and may present as erythema multiforme skin disease or skin pigmentation[19].

The histological findings and immunohistochemistry of our patients were consistent with those reported in most literature [14, 15, 20, 21], with one exception. The monomorphic and small to medium-sized tumor cells in most cases were positive for CD3, CD8, CD43, CD56, and negative for CD5, with round nuclei, obvious nucleoli, and pale cytoplasm. Increased mitotic activity, frequent necrosis, and epitheliotropic pattern could also be seen. While the outlier case showed a co-existence of cellular pleomorphism and monomorphism for the first time, which was different from the classic MEITL form. However, it still met the diagnostic criteria of MEITL. First, prominent epitheliotropic pattern and diffuse monomorphic areas could be found. Second, both monomorphic and pleomorphic cells had a typical MEITL immunophenotype of CD3+CD8+CD56+CD5-. Third, this special case also had aggressive clinical behavior and lymph node involvement. Forth, cloned T-cell receptor gene rearrangement and the genetic findings (STAT5B mutation, TP53 mutation and amplification of chr9q) detected by WES indicated that it belonged to intestinal lymphoma. In addition, this case had a longer overall survival time after CHOP chemotherapy than most patients. We hypothesized that cellular pleomorphism is related to prognosis, but due to the number of our cohort, the possibility of accidental occurrence cannot be ruled out.

WES results of nine cases showed the genetic landscape of the Chinese population. Mutation-induced abnormal activation of the JAK-STAT pathway together with chromatin remodeling and DNA damage control pathways were pervasive in diverse T cell malignancies and were reported to have an important role in oncogenesis by regulating cell proliferation, survival, differentiation, and immune response[22]. As expected, protein-coding genes involved in the JAK-STAT pathway (*STAT5B*, *JAK3*, *STAT5A*), histone modifiers (*SETD2*, *CREBBP*), and DNA damage (*TP53*) were recurrently mutated. The mutation frequencies of these genes, except *SETD2*, were similar to those in previous reports [3–5, 23].

*SETD2* is an important epigenetic gene encoding histone methyltransferase SETD2 protein which is involved in gene transcription extension and mismatch repair, and the inactivation of *SETD2* may contribute to cancer development [24]. *SETD2* was found to be the most significant recurrently mutated gene and mutated in up to 60% of patients with MEITL in previous studies reported by Tomita [5], Moffitt [4], and Roberti [3]. But in our study only 2 patients had *SETD2* mutations (2/9), showing a lower incidence. Since Tomita’s study was based on Asian patients, we ruled out the interference of ethnic heterogeneity, and we guessed it may be caused by the small sample size and environmental factors. In addition, the three *SETD2* variants were all novel and deleterious, further indicating its tumor suppressor function.

*STAT5B* was the most frequently mutated gene in our study. As a member of the STAT family, *STAT5B* plays a key regulatory role in the pathogenesis of various disease drivers, including BCR/ABL [25] and NPM-ALK [26], and expresses at a high level in hematological malignancies[27]. There have revealed multiple mutation hot-spots within SH2 and C-terminal domains in *STAT5B*, among which N642H has been discovered in various hematological malignancies of T cell origin including T-cell acute lymphoblastic leukemia(T-ALL), T-cell prolymphocytic leukemia(T-PLL), and MEITL [28]. Patient with a *STAT5B* N642H variant has a risk of relapse, drug resistance and poor outcome [29]. N642H was also detected in two of four STAT5B-mutated cases in our study and predicted to be probably damaging by Polyphen-2. Two novel variants Y665F and A776V had also been found, but needed more research to explore their functions.

Abnormalities of Chr9q were frequently detected in non-Hodgkin lymphoma, especially loss of Chr9q [30]. Both Type-I EATL and MEITL were characterized by Chr9q gains [31], and its frequency could be up to 75% in MEITL [3, 32]. Our research had verified that amplification of Chr9q(7/9) was the most common chromosome copy number variant in MEITL, and multiple important oncogenes on Chr9q showed consistent amplifications. Five cases also had mutations of JAK-STAT pathway-related genes at the same time. Though recurrent Chr9q gains were reported in mantle cell lymphoma(MCL), its frequency was lower, and the characteristic mutated genes in MCL were not related to the JAK-STAT pathway. These findings indicated that a co-occurrence of JAK-STAT pathway-related gene mutations and Chr9q gains was one of the molecular features of MEITL.

There were some limitations in the current research. First, due to the small sample size, we have not yet analyzed the correlation between the genetic changes and clinicopathological manifestations. Second, the pathogenic mechanisms of the above genetic changes on MEITL have not been further studied. We will continue to collect Chinese MEITL cases to solve the above problems and verify our results.

## Conclusions

Our work uncovers the clinicopathological and special molecular genetic characteristics of MEITL patients in the Chinese population. MEITL often occurs in elderly males and has non-specific digestive symptoms, with CD3^+^/CD8^+^/CD56^+^/CD5^−^ monomorphic medium-sized tumor cells and obvious epitheliotropic performance. Frequent mutations of *TP53*, *SETD2*and genes affecting the JAK-STAT pathway, especially *STAT5B*, are common to be discovered. The occurrence of JAK-STAT pathway-related gene mutations and the amplification of Chr9q is also a molecular feature of MEITL.

## Supplementary information

**Additional file 1**

**Additional file 2**

**Additional file 3**

## Data Availability

The datasets supporting the conclusions of this article are included within the article and its additional file. More details are not publicly available but are available from the corresponding author on reasonable request.

## Abbreviations

MEITL: Monomorphic Epitheliotropic Intestinal T-cell Lymphoma
IHC: Immunohistochemistry
WES: Whole-exome sequence
SNV: single nucleotide variant
CNV: copy number variant
GO: Gene Ontology
KEGG: Kyoto Encyclopedia of Genes and Genomes
MCL: mantle cell lymphoma
